# Dietary *Scutellaria baicalensis* extract enhances growth, intestinal health, and resistance to *Aeromonas hydrophila* in juvenile Asian swamp eel (*Monopterus albus*)

**DOI:** 10.3389/fmicb.2026.1940498

**Published:** 2026-09-18

**Authors:** Yingbing Su, Yinuo Gong, Jiali Liu, Haishan Cheng, Hongtao Liao, Ziyan Yang, Yumeng Ban, Pupu Yan, Liwei Guo, Daiqin Yang

**Affiliations:** 1 College of Animal Science and Technology, Yangtze University, Jingzhou, Hubei, China; 2 Fujian Key Laboratory of Veterinary Chinese Medicine and Animal Health, College of Animal Science, Fujian Agriculture and Forestry University, Fuzhou, Fujian, China

**Keywords:** *Aeromonas hydrophila*, growth performance, intestinal microbiota, *Monopterus albus*, *Scutellaria baicalensis* extract

## Abstract

**Introduction:**

Bacterial infection seriously restricts the healthy culture of Monopterus albus. *Scutellaria baicalensis* extract (SBE) is a natural plant‑derived additive with antioxidant and anti‑inflammatory properties. This study aimed to evaluate the effects of dietary SBE supplementation on growth performance, intestinal health, gut microbiota and resistance against *Aeromonas hydrophila* infection in juvenile Asian swamp eel.

**Methods:**

A 60‑day feeding trial was performed. Five diets were formulated with 0%, 0.5%, 1%, 2% and 3% SBE (CK, SBE‑A, SBE‑B, SBE‑C, SBE‑D). Growth indices, intestinal antioxidant status, intestinal histology, gut microbiota were detected. An *A. hydrophila* challenge test was carried out, followed by transcriptome analysis and qRT‑PCR verification of inflammation‑ and apoptosis‑related genes.

**Results:**

Supplementation with 0.5% and 1% SBE significantly improved final body weight, weight gain rate and specific growth rate, and decreased feed conversion ratio. SBE increased intestinal GSH, SOD, T‑AOC and reduced MDA; antioxidant‑related genes (*CAT*, *GSH*, *SOD*, *Keap1*) were markedly up‑regulated in the 1% SBE group. SBE optimized intestinal villus morphology, raised gut microbial diversity and the relative abundance of *Cetobacterium*, and decreased potential pathogens (*Plesiomonas*, *Brevinema*). After bacterial challenge, SBE improved survival rate, lowered tissue bacterial load and partially restored tight‑junction gene expression. Transcriptomics identified 5216 differentially expressed genes linked to inflammation, oxidative stress, metabolism and intestinal barrier. qRT‑PCR confirmed that dietary SBE regulated the expression of inflammatory and apoptotic genes induced by infection. Under current conditions, 1% dietary SBE produced the optimal beneficial effects.

**Discussion:**

In summary, dietary SBE could enhance growth performance, maintain intestinal homeostasis and improve resistance to *A. hydrophila* in juvenile *M. albus*. 1% is the recommended supplemental level in this trial.

## 1 Introduction

Aquaculture has become an increasingly important sector for providing high-quality animal protein, unsaturated fatty acids, and essential micronutrients for human consumption (Naylor et al., 2021). However, the rapid intensification of aquaculture has also increased the pressure on aquatic environments and cultured animals, leading to water quality deterioration, physiological stress, and frequent outbreaks of infectious diseases (Jia et al., 2023; Mizuta et al., 2023). In current aquaculture practice, antibiotics are still widely used for the prevention and treatment of bacterial diseases. Although antibiotics can effectively control pathogenic infections in the short term, their long-term and improper use may result in antimicrobial resistance, drug residues, food safety risks, and ecological contamination (Chen et al., 2020; Liu et al., 2017). Therefore, the development of safe, effective, and environmentally friendly feed additives has become an important strategy for improving aquatic animal health and reducing antibiotic dependence.

Medicinal plants and their bioactive extracts have attracted increasing attention as functional feed additives in aquaculture because of their growth-promoting, antioxidant, immunomodulatory, and antimicrobial properties (Reverter et al., 2014; Semwal et al., 2023). *Scutellaria baicalensis* Georgi is a traditional medicinal plant rich in flavonoids, including baicalin, baicalein, wogonin, and wogonoside, which are regarded as major active components responsible for its biological effects (Wang et al., 2018). Previous studies have shown that dietary supplementation with *S. baicalensis* extract (SBE) or its active flavonoids can improve growth performance, feed utilization, antioxidant capacity, and immune responses in several aquatic species. For example, dietary SBE improved feed utilization and challenge resistance in olive flounder (*Paralichthys olivaceus*) (Cho et al., 2013), while SBE supplementation enhanced immune-related gene expression and resistance to *Vibrio parahaemolyticus* in white shrimp (*Litopenaeus vannamei*) (Maurus et al., 2023). In addition, baicalein has been reported to alleviate liver injury in carp by modulating macrophage polarization and pyroptosis (Liu et al., 2023). These findings suggest that SBE may serve as a promising natural additive for promoting aquatic animal health. However, its effects and underlying mechanisms in Asian swamp eel (*Monopterus albus*) remain insufficiently understood.

Asian swamp eel (*M. albus*) is an economically important freshwater species widely cultured in China because of its high nutritional value and strong market demand (Hu et al., 2021). With the expansion of intensive farming systems, *M. albus* is increasingly exposed to environmental stressors and pathogenic microorganisms, which may impair intestinal health, suppress growth, and increase disease susceptibility. Among bacterial pathogens, *Aeromonas hydrophila* is one of the most important opportunistic pathogens in aquaculture and can cause hemorrhagic septicemia, tissue damage, and high mortality in fish (Abdella et al., 2024; Citterio and Francesca, 2015). The intestine is not only a major site for nutrient digestion and absorption but also an essential immune and microbial barrier against pathogen invasion. Intestinal microbiota participates in nutrient metabolism, immune regulation, epithelial barrier maintenance, and pathogen exclusion in fish (Xiong et al., 2019; Zhang et al., 2024). Therefore, nutritional strategies that improve intestinal antioxidant capacity, stabilize gut microbiota, and strengthen intestinal barrier function may help enhance resistance to *A. hydrophila* infection in *M. albus*.

Although several studies have investigated functional feed additives in *M. albus*, most have focused on growth performance, intestinal structure, or microbiota composition alone (Hu et al., 2021; Lv et al., 2024). Whether dietary SBE can simultaneously regulate growth, antioxidant defense, intestinal microbiota, barrier function, and resistance to *A. hydrophila* infection in *M. albus* has not been systematically investigated. In particular, the molecular responses associated with *A. hydrophila* infection and the possible involvement of inflammatory and apoptosis-related pathways remain unclear. Therefore, the present study evaluated the effects of dietary supplementation with different levels of SBE on growth performance, intestinal antioxidant capacity, intestinal morphology, and gut microbiota composition in juvenile *M. albus*. Furthermore, an *A. hydrophila* challenge model, bacterial load analysis, tight junction gene detection, transcriptomic analysis, PPI network construction, and qRT-PCR validation were performed to explore the protective mechanism of SBE against bacterial infection. This study aimed to provide theoretical evidence and practical guidance for the application of SBE as a functional feed additive in *M. albus* aquaculture.

## 2 Materials and methods

### 2.1 Experimental diets, fish, and feeding trial

The *Scutellaria baicalensis* extract (SBE) used in this study was purchased from Howtian Pharmaceutical Co., Ltd. (Zhucheng, China). Five experimental diets were prepared by supplementing the basal diet with 0, 0.5, 1, 2%, or 3% SBE based on feed mass, and these diets were designated as CK, SBE-A, SBE-B, SBE-C, and SBE-D, respectively. As the dietary SBE inclusion level increased, the proportion of wheat bran was correspondingly reduced to maintain the total formulation at 100%. Consequently, small differences in crude protein and crude fiber were present among the experimental diets. The composition and nutrient levels of the experimental diets are shown in Table 1.

**Table 1:** Composition and nutrient levels of the experimental diets (%).

Ingredient	CK	SBE-A	SBE-B	SBE-C	SBE-D
Fish meal	15	15	15	15	15
Fermented soybean meal	25	25	25	25	25
Corn protein powder	10	10	10	10	10
Chicken powder	10	10	10	10	10
Fish oil	5	5	5	5	5
Soybean oil	2	2	2	2	2
Wheat bran	6	5.5	4	3	2
Flour	22.7	22.7	22.7	22.7	22.7
SBE	0	0.5	1	2	3
Ca(H₂PO₄)₂	2	2	2	2	2
Choline chloride	0.25	0.25	0.25	0.25	0.25
Vitamin and mineral premix	1	1	1	1	1
Antioxidant, ethoxyquin	0.05	0.05	0.05	0.05	0.05
Total	100	100	100	100	100
Crude protein	42	41.9	41.8	41.5	41.3
Crude lipid	10	10	10.1	10.1	10.2
Ash	8.5	8.5	8.6	8.6	8.6
Crude fiber	3.5	3.7	3.8	4.0	4.2

The main nutrient levels of fish meal were 67% crude protein and 8.4% crude lipid. The premix supplied the following nutrients per kg of diet: Fe, 640 mg; Mn, 130 mg; Zn, 190 mg; vitamin C, 300 mg; Cu, 25 mg; I, 0.21 mg; Se, 0.7 mg; Co, 0.16 mg; Mg, 960 mg; K, 0.5 mg; vitamin A, 8 mg; vitamin B1, 8 mg; vitamin B2, 8 mg; vitamin B6, 12 mg; vitamin B12, 0.02 mg; vitamin D3, 3 mg; vitamin K3, 5 mg; calcium pantothenate, 25 mg; niacin, 25 mg; folic acid, 5 mg; and inositol, 100 mg.

All juvenile *Monopterus albus* were obtained from the Breeding Base of Yangtze University (Jingzhou, China). Before the feeding trial, healthy and active fish were selected and acclimated in breeding net cages (100 cm × 80 cm × 120 cm) for 7 days. After acclimation, 300 fish were randomly allocated into five dietary groups, with 60 fish per group and three replicates per group. The feeding trial lasted for 60 days. During the experiment, fish were fed once daily at 18:00, and the feeding rate was adjusted to approximately 5% of body weight by weighing fish every 7 days. Uneaten feed and feces were removed daily.

### 2.2 Sample collection

After the 60-day feeding trial, fish were fasted for 24 h and anesthetized with MS-222. Body weight was recorded before sampling. Fish were dissected on ice, and distal intestinal tissues were carefully collected. Part of the intestinal tissue was fixed in 4% paraformaldehyde for histological analysis. The remaining intestinal samples were immediately frozen in liquid nitrogen and stored at −80 °C for antioxidant enzyme assays, qRT-PCR analysis, and intestinal microbiota sequencing. The sampling procedure followed animal welfare requirements and was approved by the Animal Ethics Committee of Yangtze University (YJ 202344).

### 2.3 Growth performance calculation

Growth performance was evaluated based on initial body weight (IBW), final body weight (FBW), weight gain rate (WGR), specific growth rate (SGR), and feed conversion ratio (FCR). The formulas were as follows:

WGR(%)=100×(FBW−IBW)/IBW

SGR(%/day)=100×[ln(FBW)−ln(IBW)]/feeding days

FCR=feed intake/body weight gain

### 2.4 Histological analysis of intestinal tissue

Distal intestinal tissues from five fish per treatment were fixed in 4% paraformaldehyde, dehydrated through a graded ethanol series, cleared with xylene, and embedded in paraffin. Sections with a thickness of 5 μm were prepared using a rotary microtome, deparaffinized, rehydrated, and stained with hematoxylin and eosin (H&E). For each fish, five sections were examined under a light microscope, and ten well-oriented villi were measured per section using ImageJ software. Only intact villi with a clearly identifiable base and tip, a continuous epithelial structure, and a longitudinal orientation were included. Obliquely sectioned, folded, damaged, or incompletely visualized villi were excluded. Villus measurements from each fish were averaged to obtain one biological replicate, and five fish per treatment were used for statistical analysis.

### 2.5 Determination of intestinal antioxidant parameters

Frozen intestinal tissues were thawed on ice. Approximately 0.1 g of intestinal tissue was weighed and homogenized with 0.9 mL of pre-cooled phosphate-buffered saline (PBS) to prepare a 10% tissue homogenate. The homogenates were centrifuged at 3,000 rpm for 10 min at 4 °C, and the supernatants were collected for biochemical analysis.

The levels of malondialdehyde (MDA, A003-1-2), superoxide dismutase (SOD, A001-3-2), total antioxidant capacity (T-AOC), and reduced glutathione (GSH, A005-1-2) were determined using commercial assay kits purchased from Nanjing Jiancheng Bioengineering Institute (Nanjing, China), according to the manufacturer’s instructions.

### 2.6 RNA extraction, reverse transcription, and qRT-PCR

Total RNA was extracted from intestinal tissues using the FastPure Cell/Tissue Total RNA Isolation Kit (Vazyme Biotech Co., Ltd., Nanjing, China), according to the manufacturer’s instructions. RNA concentration and purity were determined using a NanoDrop spectrophotometer (NanoDrop Technologies, USA) by measuring the absorbance ratio at 260/280 nm. RNA integrity was verified by 1% agarose gel electrophoresis.

For reverse transcription, qualified RNA was reverse-transcribed into cDNA using a commercial reverse transcription kit. The reaction mixture was prepared according to the manufacturer’s instructions. The synthesized cDNA was diluted and stored at −20 °C until qRT-PCR analysis.

qRT-PCR was performed using a SYBR Green-based quantitative PCR system on a LineGene 9,600 Plus Fluorescent Quantitative PCR Detection System (Bioer Technology, Hangzhou, China). Each 20 μL qRT-PCR reaction contained 10 μL of 2 × SYBR qPCR Master Mix, 0.4 μL of forward primer, 0.4 μL of reverse primer, 1 μL of cDNA template, and 8.2 μL of nuclease-free water. The amplification program was as follows: initial denaturation at 95 °C for 30 s, followed by 40 cycles of 95 °C for 10 s and 60 °C for 30 s. Amplification specificity was assessed by melting-curve analysis, and a single predominant melting peak was observed for each primer pair. Each sample was analyzed in triplicate. β-actin was used as the internal reference gene, and the relative mRNA expression levels of target genes were calculated using the 2^−ΔΔCt^ method.

Target genes included antioxidant-related genes (*CAT*, *GSH*, *SOD*, and *Keap1*), immune-related genes (*IFN-γ*, *NF-κB*, *TLR4*, *IL-1β*, *IL-6*, and *TNF-α*), apoptosis-related genes (*Caspase-3*, *Caspase-9*, and *Bcl-2*), and hub genes selected from transcriptomic and PPI analyses. The confirmed primer sequences are listed in Table 2.

**Table 2:** Primer sequences used for qRT-PCR in *M. albus*.

Gene	Primer sequence (5′–3′)	GenBank accession no.
*CAT*	F: CACCTATCGCTGTCCG R: CCAGTTGCCATCCTCA	NW_018127887.1
*SOD*	F: CAGGACCTCACTTCAATC R: CTCCACAAGCCAGACG	NW_018127918.1
*Keap1*	F: CTGGAGCGTCAGTTGTG R: GACCCTGGGATGGATT	NW_018127912.1
*GSH*	F: CTGGGATGCTCGTCTT R: TTACCTCCTCCTTCTCG	XM_020617951.1
*IFN-γ*	F: TCGTCCTGGTTCTCCTC R: CGTGGCTCACTTTATCG	XM_035401561.1
*SRC*	F: GCCTCACAGATTGCTT R: TTGATGGGAAACTTGG	NW_018128092.1
*JAK1*	F: TGGGCACAAGAACTAACA R: TCCATACAGCAGCACAAT	NW_018127938.1
*STAT3*	F: AGTGCGTGCATCGTAT R: TGTAGGCTGCGGTTTA	NW_018127965.1
*TP53*	F: CCAGTTACATCCGACTACCC R: CCCTGATAATGGCACCCT	NW_018128264.1
*SIRT1*	F: TTGACGGGCTGGAAAGAT R: GGAACCGAACTACGCACA	NW_018128108.1
*NF-κB*	F: AGGTTTCGCTATGAATGTG R: GTGTTTGGGCAGTCTTTAC	NW_018128208.1
*TLR4*	F: CAGAACTGACCCACCATC R: CCACGGGCATAAACATAG	NW_018128023.1
*IL-6*	F: GCAACTGCTGAGGGAC R: GCCGCACTTCTTCATT	NW_018127962.1
*IL-1β*	F: CCTCATTATCGCCACG R: TCGCACCATATCAGTTTC	NW_018128256.1
*TNF-α*	F: ATGGATACAGGCTCAACAG R: AGAGGAACTTCAGCGTCA	NW_018128146.1
*Caspase-3*	F: AGACTCCACCGCACCCTCA R: GCTGGCGATTGCCTTCCT	NW_018128248.1
*Caspase-9*	F: CAGACAGATGCCATACCG R: TCGCAACATACCAGGAGC	NW_018128006.1
*Bcl-2*	F: CAGAGTGGGCTGGGATG R: TTGCGGACAAAGTGGC	NW_018127908.1
*β-actin*	F: CCTGTATGCCTCTGGT R: TAATGTCACGCACGAT	NW_018128168.1

### 2.7 Intestinal microbiota sequencing and bioinformatics analysis

Genomic DNA was extracted from intestinal samples using a modified SDS-based method. DNA purity and concentration were assessed by agarose gel electrophoresis. The V3–V4 hypervariable region of the bacterial 16S rRNA gene was amplified using the forward primer 341F (5′-CCTAYGGGRBGCASCAG-3′) and reverse primer 806R (5′-GGACTACNNGGGTATCTAAT-3′). PCR amplicons were purified using AMPure XP magnetic beads (Beckman Coulter, Indianapolis, IN, USA) and quantified using the Qubit dsDNA Assay Kit (Invitrogen, Carlsbad, CA, USA). Sequencing libraries were constructed using the SMRTbell Express Template Prep Kit 2.0-SPv4 (Pacific Biosciences, Menlo Park, CA, USA), and high-throughput sequencing was performed on the PacBio Sequel II platform.

Raw sequencing data were processed using the DADA2 R package. Quality filtering, denoising, chimera removal, and amplicon sequence variant (ASV) generation were performed. Taxonomic annotation was conducted using the SILVA database. Alpha diversity indices, including Chao1, Shannon, and Simpson, were calculated. Principal coordinate analysis (PCoA) and non-metric multidimensional scaling (NMDS) were performed to evaluate differences in microbial community structure among groups. Additional analyses, including sample correlation analysis, differential taxa analysis, functional prediction, and microbial co-occurrence network analysis, were performed to further characterize the effect of SBE on intestinal microbiota.

### 2.8 *Aeromonas hydrophila* culture and challenge test

*Aeromonas hydrophila* ATCC7966 was provided by the Laboratory of Animal Pathogenic Microbiology, College of Animal Science and Technology, Yangtze University. The bacterial strain stored at −80 °C was thawed at room temperature. Then, 100 μL of bacterial suspension was inoculated into 5 mL of LB liquid medium and cultured at 28 °C with shaking at 180 rpm. When the OD600 reached approximately 0.6, the bacterial culture was streaked onto LB agar plates and incubated overnight at 28 °C for purification. A single colony was selected and transferred into fresh LB liquid medium for secondary activation. When the OD600 reached approximately 0.6 again, the bacterial suspension was collected and diluted with sterile PBS to a final concentration of 2 × 10^8^ CFU/mL for the challenge test.

After the 60-day feeding trial, the *A. hydrophila* challenge test was performed to evaluate the protective effect of dietary SBE supplementation against bacterial infection. The challenge experiment included six groups: CK, Model, SBE-A, SBE-B, SBE-C, and SBE-D. For each group, 20 fish were used for the 72 h survival analysis, and an additional six fish were assigned for tissue collection at 24 h post-infection. Fish in the CK group were intraperitoneally injected with 100 μL of sterile PBS and served as the uninfected control. Fish in the Model group and the SBE-supplemented groups were intraperitoneally injected with 100 μL of *A. hydrophila* suspension at 2 × 10^8^ CFU/mL, corresponding to an inoculum of 2 × 10^7^ CFU per fish. This challenge condition produced a severe acute infection and was therefore interpreted as a stringent lethal challenge rather than a moderate infection model. Survival status and clinical signs were recorded during the subsequent 72 h observation period.

### 2.9 Determination of culturable bacterial burden in tissues

To evaluate early tissue bacterial dissemination after *A. hydrophila* challenge, culturable bacterial burdens were determined at 24 h post-infection. This time point was used to assess bacterial dissemination before extensive mortality occurred in the severely challenged Model group, thereby reducing potential survivorship bias and post-mortem bacterial proliferation. Six fish were randomly selected from each infected group for tissue collection. The intestine, spleen, and liver were aseptically dissected, weighed, and homogenized in sterile PBS. The tissue homogenates were serially diluted, and 100 μL of each dilution was spread onto LB agar plates. After incubation at 28 °C until visible colonies formed, colonies were counted, and the culturable bacterial burden was expressed as CFU/g tissue. Because LB agar is non-selective and species-specific identification of individual colonies was not performed, these plate counts were interpreted as culturable bacterial burden rather than species-specific quantification of *A. hydrophila*. During the 72 h survival observation, dead fish were recorded and removed immediately. These dead fish were used only for survival analysis and were not included in the bacterial burden analysis to minimize potential bias associated with differences in death time and post-mortem bacterial proliferation.

### 2.10 Transcriptome sequencing and differential expression analysis

To investigate the intestinal molecular response of *M. albus* to *A. hydrophila* infection, intestinal tissues from the CK and Model groups were collected for transcriptome sequencing. Total RNA was extracted from intestinal tissues. RNA quality and integrity were assessed before library construction. Qualified RNA samples were used to construct sequencing libraries.

Raw sequencing data were filtered to obtain clean reads. Clean reads were mapped to the reference genome or transcriptome of *M. albus*. Gene expression levels were calculated using FPKM values. Differentially expressed genes (DEGs) between the CK and Model groups were identified using false discovery rate (FDR) < 0.05 and |log2 fold change| > 1 as thresholds. GO and KEGG enrichment analyses were performed to identify biological functions and pathways associated with DEGs.

### 2.11 PPI network construction and hub gene screening

DEGs identified between the CK and Model groups were imported into the STRING database for protein–protein interaction (PPI) analysis. After removing proteins without interaction relationships, the PPI network was visualized using Cytoscape software. Hub genes within the infection-responsive network were screened using the CytoHubba plugin according to degree values. Selected infection-responsive hub genes were subsequently examined by qRT-PCR across the CK, Model, and SBE-treated groups to determine whether dietary SBE was associated with modulation of their transcription. The thesis lists the top hub genes as *TP53*, *SRC*, *AKT1*, *GAPDH*, *STAT3*, *SIRT1*, *MET*, *JAK1*, *MTOR*, and *ERBB2*.

### 2.12 Statistical analysis

All data are presented as mean ± standard error (SE). Statistical analyses were performed using SPSS 27.0 and GraphPad Prism 9.0. Before statistical comparison, quantitative data were tested for normality and homogeneity of variance. Differences among groups were analyzed using one-way analysis of variance (ANOVA), followed by Tukey’s multiple-comparison test for multiple comparisons. Survival following *A. hydrophila* challenge was analyzed using the Kaplan–Meier method, and differences among survival curves were evaluated using the log-rank (Mantel–Cox) test. A value of *p* < 0.05 was considered statistically significant.

## 3 Results

### 3.1 Dietary SBE supplementation improved growth performance of juvenile *M. albus*

No disease or mortality was observed during the 60-day feeding trial. As shown in Table 3, there was no significant difference in initial body weight among the CK, SBE-A, SBE-B, SBE-C, and SBE-D groups (*p* > 0.05), indicating that the experimental fish had comparable starting conditions. After 60 days of feeding, dietary SBE supplementation significantly affected the final body weight of juvenile *M. albus*. Compared with the CK group, the final body weight was significantly increased in the SBE-A, SBE-B, and SBE-C groups (*p* < 0.05), with the highest value observed in the SBE-B group. Specifically, the final body weight increased from 46.40 ± 1.11 g in the CK group to 55.92 ± 0.93 g and 60.80 ± 2.14 g in the SBE-A and SBE-B groups, respectively. The SBE-B group also showed a significantly higher final body weight than the other SBE-supplemented groups (*p* < 0.05), indicating that 1% SBE exerted the strongest growth-promoting effect.

**Table 3:** Effects of dietary *Scutellaria baicalensis* extract supplementation on the growth performance of juvenile *Monopterus albus*.

Indicators	Groups				
	CK	SBE-A	SBE-B	SBE-C	SBE-D
IBW/g	10.66 ± 0.27^a^	11.34 ± 0.22^a^	11.24 ± 0.40^a^	10.86 ± 0.33^a^	10.76 ± 0.25^a^
FBW/g	46.40 ± 1.11^d^	55.92 ± 0.93^b^	60.80 ± 2.14^a^	50.48 ± 1.38^c^	49.42 ± 1.18^cd^
WGR/%	76.97 ± 1.79^b^	79.72 ± 1.22^a^	81.43 ± 2.01^a^	78.36 ± 2.67^ab^	78.23 ± 1.95^ab^
SGR/(%/d)	2.45 ± 0.06^c^	2.66 ± 0.01^ab^	2.81 ± 0.08^a^	2.56 ± 0.09^bc^	2.54 ± 0.03^bc^
FCR	2.23 ± 0.63^c^	2.02 ± 0.38^ab^	1.92 ± 0.83^a^	2.02 ± 0.58^ab^	2.07 ± 0.56^b^

Values are expressed as mean ± SE. Different superscript letters within the same row indicate significant differences among groups (*p* < 0.05). IBW, initial body weight; FBW, final body weight; WGR, weight gain rate; SGR, specific growth rate; FCR, feed conversion ratio. CK: basal diet group; SBE-A: 0.5% SBE group; SBE-B: 1% SBE group; SBE-C: 2% SBE group; SBE-D: 3% SBE group.

Consistent with the changes in body weight, the weight gain rate and specific growth rate were significantly increased in the SBE-A and SBE-B groups compared with the CK group (*p* < 0.05). Meanwhile, the feed conversion ratio was significantly reduced in the SBE-supplemented groups compared with the CK group (*p* < 0.05), with the lowest value observed in the SBE-B group. Although the SBE-C and SBE-D groups showed lower FCR values than the CK group, their FBW, WGR, and SGR did not show corresponding significant improvements. These findings indicate that the responses of feed utilization and growth performance were not fully parallel across SBE inclusion levels. Overall, the 1% SBE group showed the most consistently favorable response under the present experimental conditions.

### 3.2 Dietary SBE supplementation improved intestinal antioxidant status and morphology

As shown in Figures 1A–D, dietary SBE supplementation markedly affected the intestinal antioxidant status of juvenile *M. albus*. Compared with the CK group, the levels of GSH and SOD were significantly increased in all SBE-supplemented groups, with the highest values observed in the SBE-B group (*p* < 0.05). Similarly, T-AOC was significantly elevated in the SBE-A and SBE-B groups compared with the CK group, whereas no significant improvement was observed in the high-dose SBE groups. In contrast, intestinal MDA content was significantly decreased after SBE supplementation, particularly in the SBE-B group, indicating that 1% SBE had the strongest inhibitory effect on lipid peroxidation.

**Figure 1:**
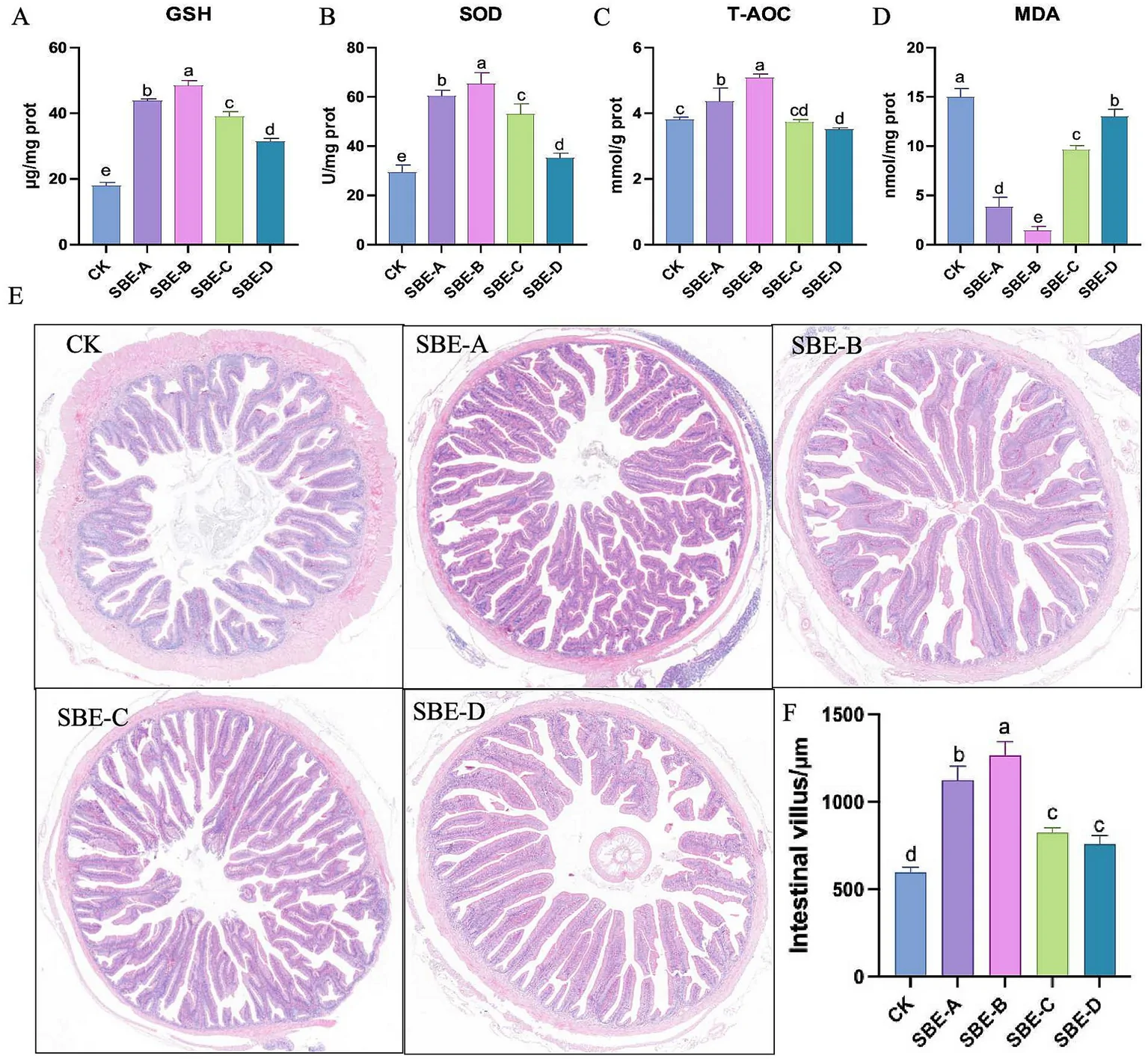
Effects of dietary SBE supplementation on intestinal antioxidant capacity and morphology in juvenile *Monopterus albus*. **(A)** GSH level. **(B)** SOD activity. **(C)** T-AOC level. **(D)** MDA content. **(E)** Representative H&E-stained intestinal sections from the CK, SBE-A, SBE-B, SBE-C, and SBE-D groups. **(F)** Quantitative analysis of intestinal villus length. Values are expressed as mean ± SE. Different letters indicate significant differences among groups (*p* < 0.05). CK: basal diet group; SBE-A: 0.5% SBE group; SBE-B: 1% SBE group; SBE-C: 2% SBE group; SBE-D: 3% SBE group.

Histological analysis further showed that SBE supplementation improved intestinal morphology (Figure 1E). The intestinal villi in the CK group were relatively short and loosely arranged, whereas the SBE-A and SBE-B groups exhibited longer villi, more regular villus structures, and a more compact mucosal architecture. Quantitative analysis confirmed that villus length was significantly increased in the SBE-treated groups compared with the CK group, with the greatest increase observed in the SBE-B group (Figure 1F). However, villus length in the SBE-C and SBE-D groups was significantly lower than that in the SBE-A and SBE-B groups. These results suggest that appropriate SBE supplementation improved intestinal antioxidant capacity and mucosal morphology in *M. albus*, with 1% SBE showing the most pronounced effect.

### 3.3 Dietary SBE supplementation regulated antioxidant and immune related gene expression

As shown in Figure 2, dietary SBE supplementation markedly affected the intestinal expression of antioxidant- and immune-related genes in juvenile *M. albus*. Compared with the CK group, the mRNA expression levels of *CAT*, *GSH*, *SOD*, and *Keap1* were significantly upregulated in the SBE-B group (*p* < 0.05), indicating that 1% SBE effectively enhanced the intestinal antioxidant defense system. Among these genes, *GSH* and *Keap1* expression was also significantly increased in the SBE-A group, whereas no significant increase was observed in the SBE-C and SBE-D groups. In contrast, the expression levels of *CAT* and *SOD* tended to decline in the high-dose SBE-D group.

**Figure 2:**
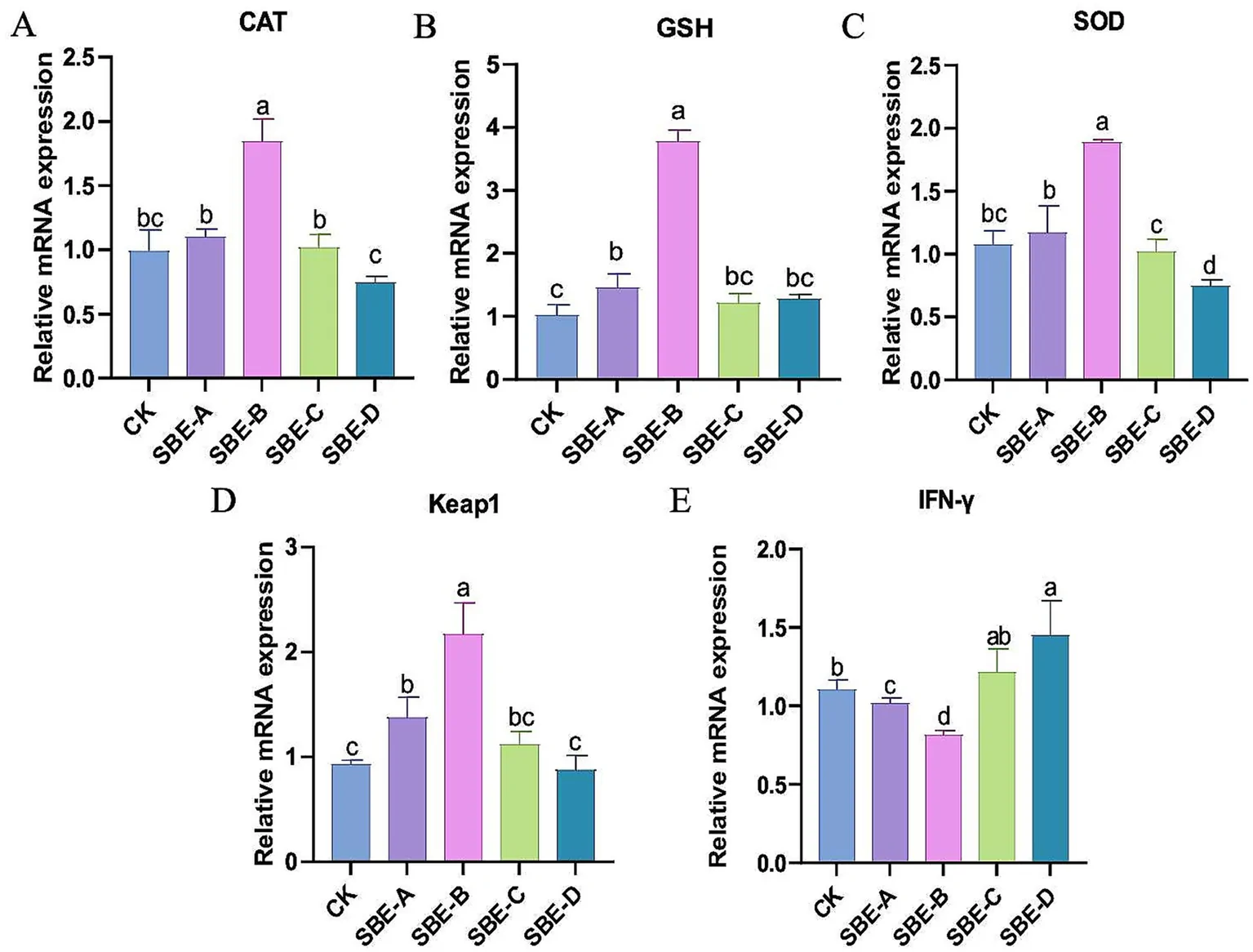
Effects of dietary SBE supplementation on antioxidant- and immune-related gene expression in the intestine of juvenile *Monopterus albus*. **(A)** Relative mRNA expression of CAT. **(B)** Relative mRNA expression of GSH. **(C)** Relative mRNA expression of SOD. **(D)** Relative mRNA expression of Keap1. **(E)** Relative mRNA expression of IFN-γ. Values are expressed as mean ± SE. Different letters indicate significant differences among groups (*p* < 0.05). CK: asal diet group; SBE-A: 0.5% SBE group; SBE-B: 1% SBE group; SBE-C: 2% SBE group; SBE-D: 3% SBE group.

For the immune-related gene, *IFN-γ* expression was significantly decreased in the SBE-A and SBE-B groups compared with the CK group, with the lowest level observed in the SBE-B group. However, *IFN-γ* expression was significantly increased in the SBE-D group. These results suggest that dietary supplementation with 1% SBE enhanced antioxidant-related gene expression while suppressing inflammatory signaling in the intestine of *M. albus*.

### 3.4 Dietary SBE supplementation reshaped intestinal microbiota diversity and community structure

As shown in Figures 3A–C, dietary SBE supplementation affected the alpha diversity of the intestinal microbiota in juvenile *M. albus*. Compared with the CK group, the Chao1 and Shannon indices were significantly increased in the SBE-B group, indicating that 1% SBE supplementation enhanced both microbial richness and diversity. The Simpson index also showed a higher level in the SBE-B group than in the CK group. In contrast, the alpha diversity indices decreased in the SBE-D group, suggesting that excessive SBE supplementation may weaken its beneficial regulatory effect on intestinal microbial diversity.

**Figure 3:**
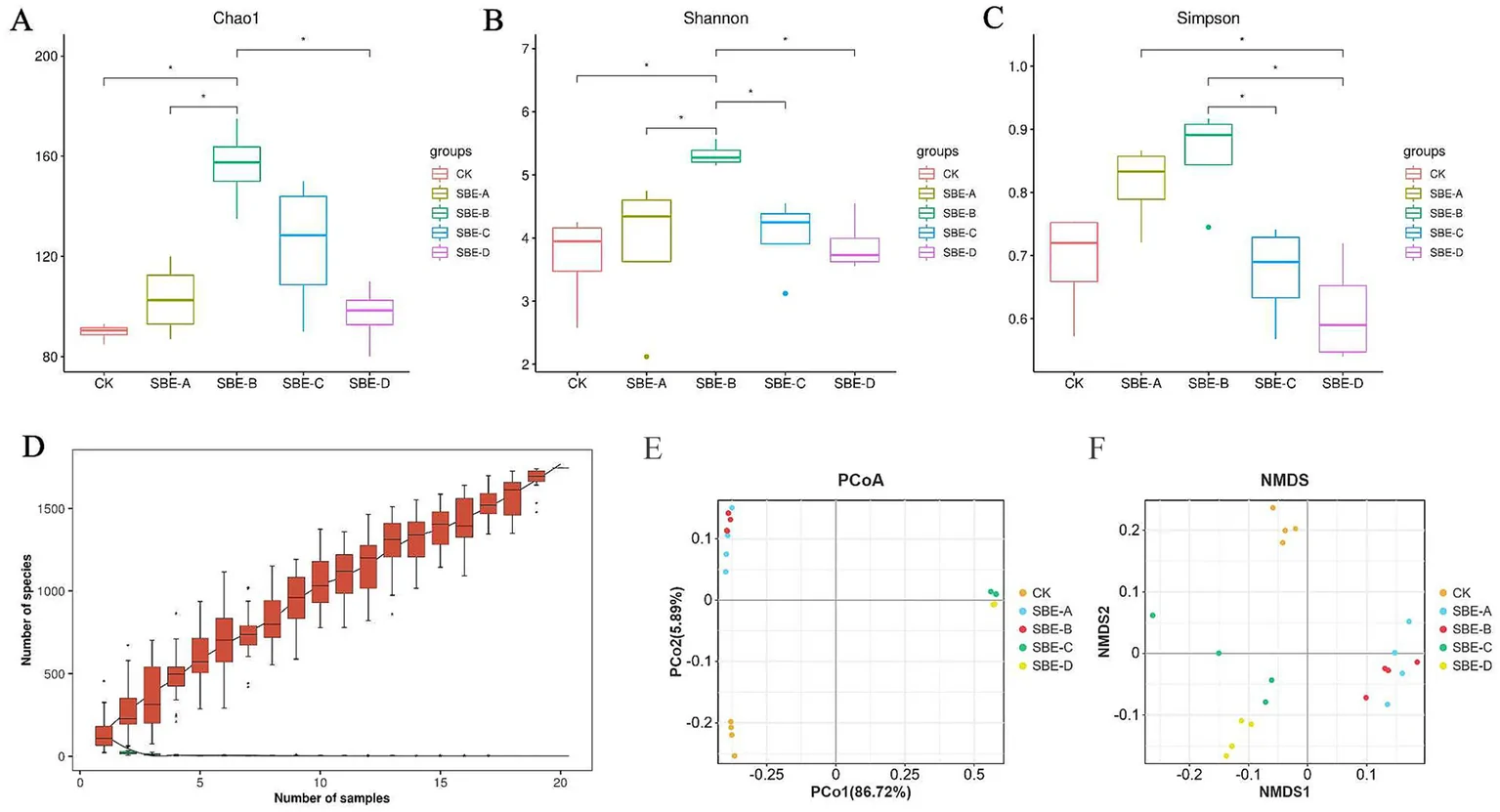
Effects of dietary SBE supplementation on intestinal microbiota diversity and community structure in juvenile *Monopterus albus*. **(A)** Chao1 index. **(B)** Shannon index. **(C)** Simpson index. **(D)** Species accumulation curve. **(E)** Principal coordinate analysis (PCoA) based on Bray–Curtis distance. **(F)** Non-metric multidimensional scaling (NMDS) analysis. Asterisks indicate significant differences among groups (*p* < 0.05). CK: asal diet group; SBE-A: 0.5% SBE group; SBE-B: 1% SBE group; SBE-C: 2% SBE group; SBE-D: 3% SBE group.

The species accumulation curve tended to reach a plateau as the number of samples increased, indicating that the sequencing depth was sufficient to reflect the intestinal microbial composition of *M. albus* (Figure 3D). Beta diversity analysis further showed that dietary SBE supplementation altered the intestinal microbial community structure. In the PCoA plot, the CK group was clearly separated from the SBE-supplemented groups, with the first two principal coordinates explaining 86.72 and 5.89% of the total variation, respectively (Figure 3E). Similarly, NMDS analysis showed distinct clustering patterns among different dietary treatments (Figure 3F).

To further confirm the effect of SBE on intestinal microbial structure, extended microbiota analyses were performed. Consistent with the main beta-diversity results, the PCoA and NMDS plots in Supplementary Figures 1A and B showed clear separation between the CK group and the SBE-supplemented groups. The sample correlation heatmap indicated good consistency among biological replicates within each group (Supplementary Figure 1C). Differential taxa analysis showed that SBE supplementation altered several bacterial taxa associated with intestinal microbial balance (Supplementary Figure 1D). In addition, predicted functional profiles and microbial co-occurrence network analysis suggested that SBE may influence not only bacterial composition but also potential microbial metabolic functions and ecological interactions within the intestinal community (Supplementary Figures 1E–G). Collectively, these results indicate that dietary SBE supplementation reshaped the intestinal microbiota of *M. albus*.

### 3.5 Dietary SBE supplementation altered intestinal microbiota composition

As shown in Figure 4A, the intestinal microbiota of juvenile *M. albus* was mainly composed of *Desulfobacterota*, *Firmicutes*, *Proteobacteria*, *Spirochaetota*, *Fusobacteriota*, *Bacteroidota*, *Actinobacteriota*, *Planctomycetota*, *Verrucomicrobiota*, and *Cyanobacteria* at the phylum level. Compared with the CK group, dietary SBE supplementation significantly altered the relative abundance of several dominant bacterial phyla. The relative abundance of *Firmicutes* was significantly increased in the SBE-B group compared with the CK group (*p* < 0.05), whereas it was significantly decreased in the SBE-C and SBE-D groups (*p* < 0.05; Figure 4B). In contrast, *Proteobacteria* was markedly reduced in all SBE-supplemented groups compared with the CK group (*p* < 0.05), with the lowest abundance observed in the SBE-A and SBE-B groups (Figure 4C). The relative abundance of *Bacteroidota* was significantly increased in the SBE-A and SBE-B groups compared with the CK group (*p* < 0.05), particularly in the SBE-B group (Figure 4D). Moreover, the *Firmicutes/Bacteroidota* ratio was significantly decreased after SBE supplementation, especially in the SBE-A and SBE-B groups (*p* < 0.05; Figure 4E).

**Figure 4:**
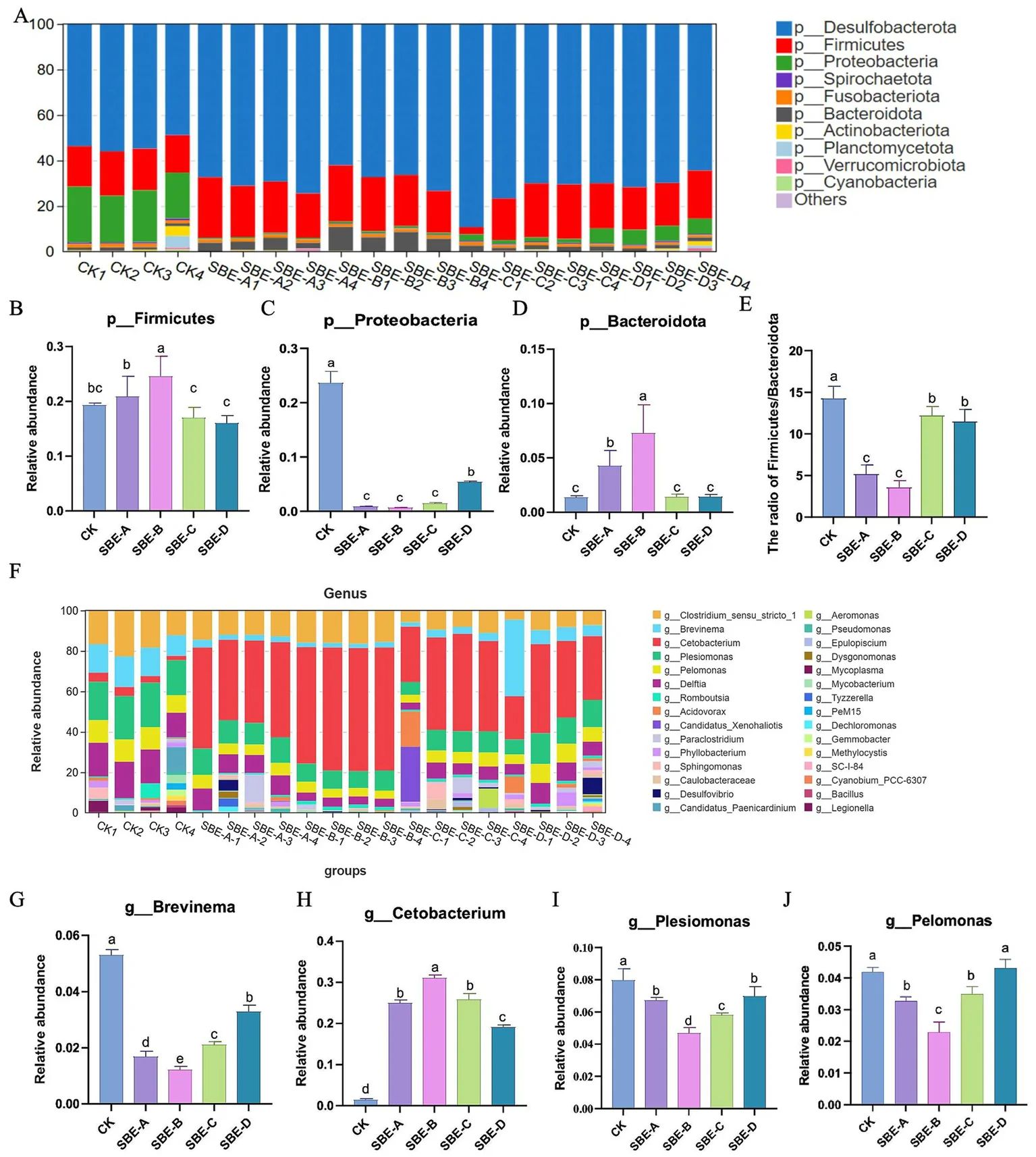
Effects of dietary SBE supplementation on intestinal microbiota composition in juvenile *Monopterus albus*. **(A)** Relative abundance of the top 10 bacterial phyla. **(B)** Relative abundance of *Firmicutes*. **(C)** Relative abundance of *Proteobacteria*. **(D)** Relative abundance of *Bacteroidota*. **(E)** Firmicutes/Bacteroidota ratio. **(F)** Relative abundance of dominant bacterial genera. **(G)** Relative abundance of *Brevinema*. **(H)** Relative abundance of *Cetobacterium*. **(I)** Relative abundance of *Plesiomonas*. **(J)** Relative abundance of *Pelomonas*. Values are expressed as mean ± SE. Different letters indicate significant differences among groups (*p* < 0.05). CK: Basal diet group; SBE-A: 0.5% SBE group; SBE-B: 1% SBE group; SBE-C: 2% SBE group; SBE-D: 3% SBE group.

At the genus level, the dominant genera included *Clostridium_sensu_stricto_1*, *Brevinema*, *Cetobacterium*, *Plesiomonas*, *Pelomonas*, *Delftia*, *Romboutsia*, and other bacterial taxa (Figure 4F). Compared with the CK group, the relative abundance of *Brevinema* was significantly decreased in all SBE-supplemented groups (*p* < 0.05), with the lowest level observed in the SBE-B group (Figure 4G). In contrast, the relative abundance of *Cetobacterium* was markedly increased after SBE supplementation (*p* < 0.05), and the highest abundance was observed in the SBE-B group (Figure 4H). The relative abundance of *Plesiomonas* was also significantly reduced in the SBE-supplemented groups compared with the CK group (*p* < 0.05), particularly in the SBE-B group (Figure 4I). Similarly, *Pelomonas* abundance was significantly decreased in the SBE-A, SBE-B, and SBE-C groups compared with the CK group (*p* < 0.05), with the lowest level detected in the SBE-B group (Figure 4J). These results indicate that dietary SBE supplementation significantly improved the intestinal microbial composition of *M. albus* by reducing potential pathogenic bacteria and enriching beneficial bacteria.

### 3.6 Dietary SBE supplementation enhanced resistance to *A. hydrophila* infection

As shown in Figure 5A, *A. hydrophila* challenge markedly reduced the survival rate of juvenile *M. albus.* The CK group maintained 100% survival throughout the observation period, whereas the survival rate of the Model group rapidly declined and reached 0% at 60 h post-infection. Compared with the Model group, dietary SBE supplementation improved the survival of infected *M. albus*, indicating that SBE enhanced host resistance to *A. hydrophila* infection. Among the SBE-treated groups, the SBE-B group showed the highest survival rate after challenge, indicating that the 1% SBE group showed the most favorable survival outcome among the tested inclusion levels under the present experimental conditions.

**Figure 5:**
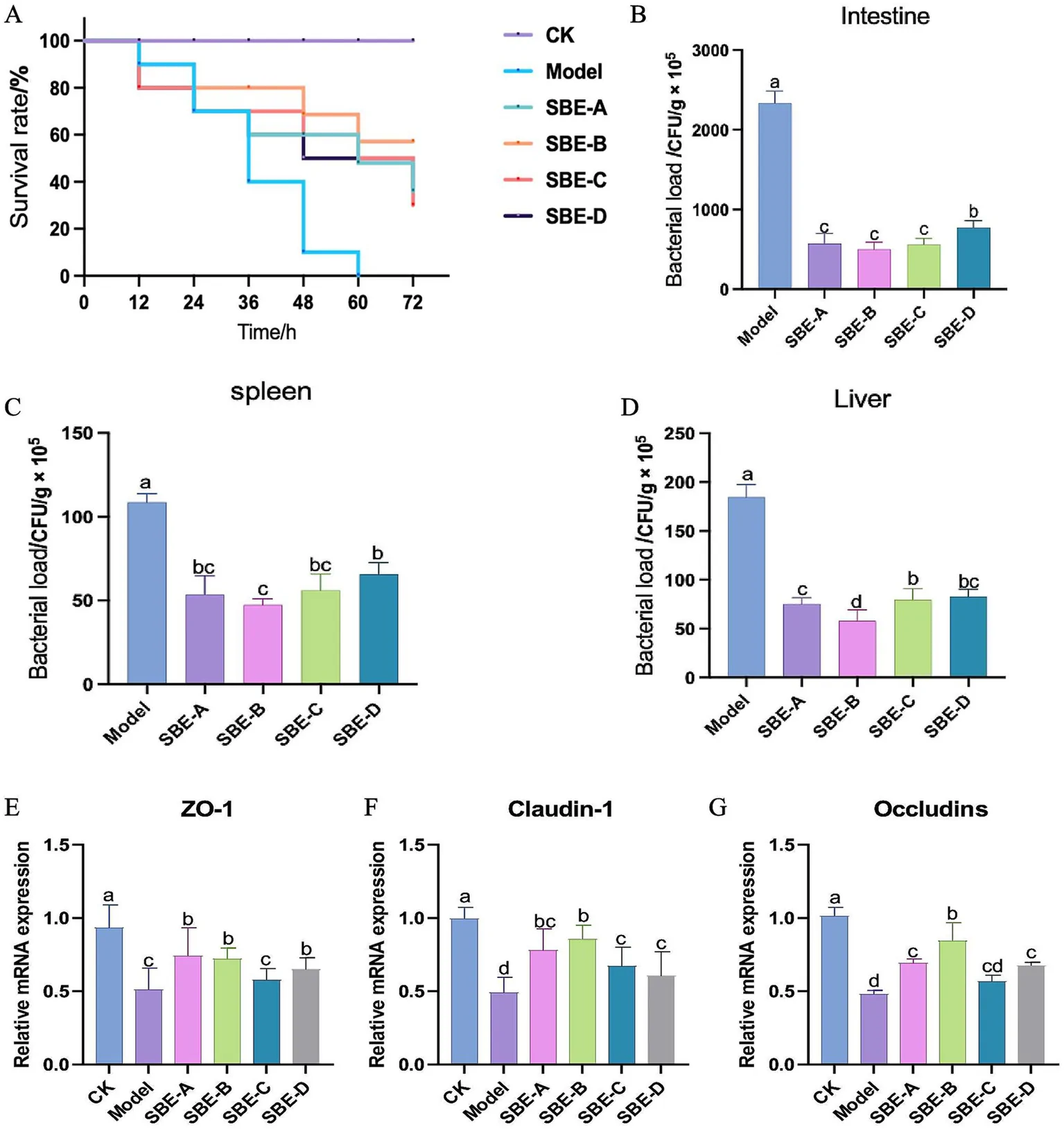
Effects of dietary SBE supplementation on resistance to *Aeromonas hydrophila inf*ection and intestinal barrier-related gene expression in juvenile *Monopterus albus*. **(A)** Kaplan–Meier survival curves of *M. albus* following *A. hydrophila* challenge. **(B)** Culturable bacterial burden in the intestine. **(C)** Culturable bacterial burden in the spleen. **(D)** Culturable bacterial burden in the liver. **(E)** Relative mRNA expression of *ZO-1*. **(F)** Relative mRNA expression of *Claudin-1*. **(G)** Relative mRNA expression of *Occludin*. Values are expressed as mean ± SE. Different letters indicate significant differences among groups (*p* < 0.05). CK: uninfected control group; Model: *A. hydrophila*-infected group; SBE-A: 0.5% SBE group after infection; SBE-B: 1% SBE group after infection; SBE-C: 2% SBE group after infection; SBE-D: 3% SBE group after infection.

Compared with the Model group, all SBE-supplemented groups showed significantly lower culturable bacterial burdens in the intestine, spleen, and liver (Figures 5B–D). In particular, the SBE-B group exhibited the lowest bacterial burden in these tissues, indicating that 1% SBE was associated with the lowest culturable bacterial burden in the examined tissues after *A. hydrophila* challenge.

The expression of intestinal tight junction-related genes was also examined after *A. hydrophila* challenge. Compared with the CK group, the mRNA expression levels of *ZO-1*, *Claudin-1*, and *Occludin* were significantly downregulated in the Model group, indicating a marked transcriptional disturbance in tight junction-related genes (Figures 5E–G). Dietary SBE supplementation partially restored the expression of these genes. Among the SBE groups, SBE-A and SBE-B showed stronger effects on *ZO-1* expression, whereas SBE-B showed the most pronounced changes in *Claudin-1* and *Occludin* expression. Together, these results indicate that SBE supplementation was associated with reduced culturable bacterial burden and partial recovery of tight junction-related gene expression following *A. hydrophila* challenge, with the most favorable response observed in the 1% SBE group under the present experimental conditions.

### 3.7 Transcriptomic analysis revealed intestinal molecular responses to *A. hydrophila* infection

To investigate the intestinal molecular responses of *M. albus* to *A. hydrophila* infection, transcriptome sequencing was performed between the CK and Model groups. As shown in Figure 6A, the biological replicates within each group showed high correlation, while the CK and Model groups were clearly separated, indicating good reproducibility and distinct transcriptional profiles between the two groups. The distribution of gene expression levels was generally consistent among samples, suggesting that the sequencing data were reliable for subsequent differential expression analysis (Figure 6B).

**Figure 6:**
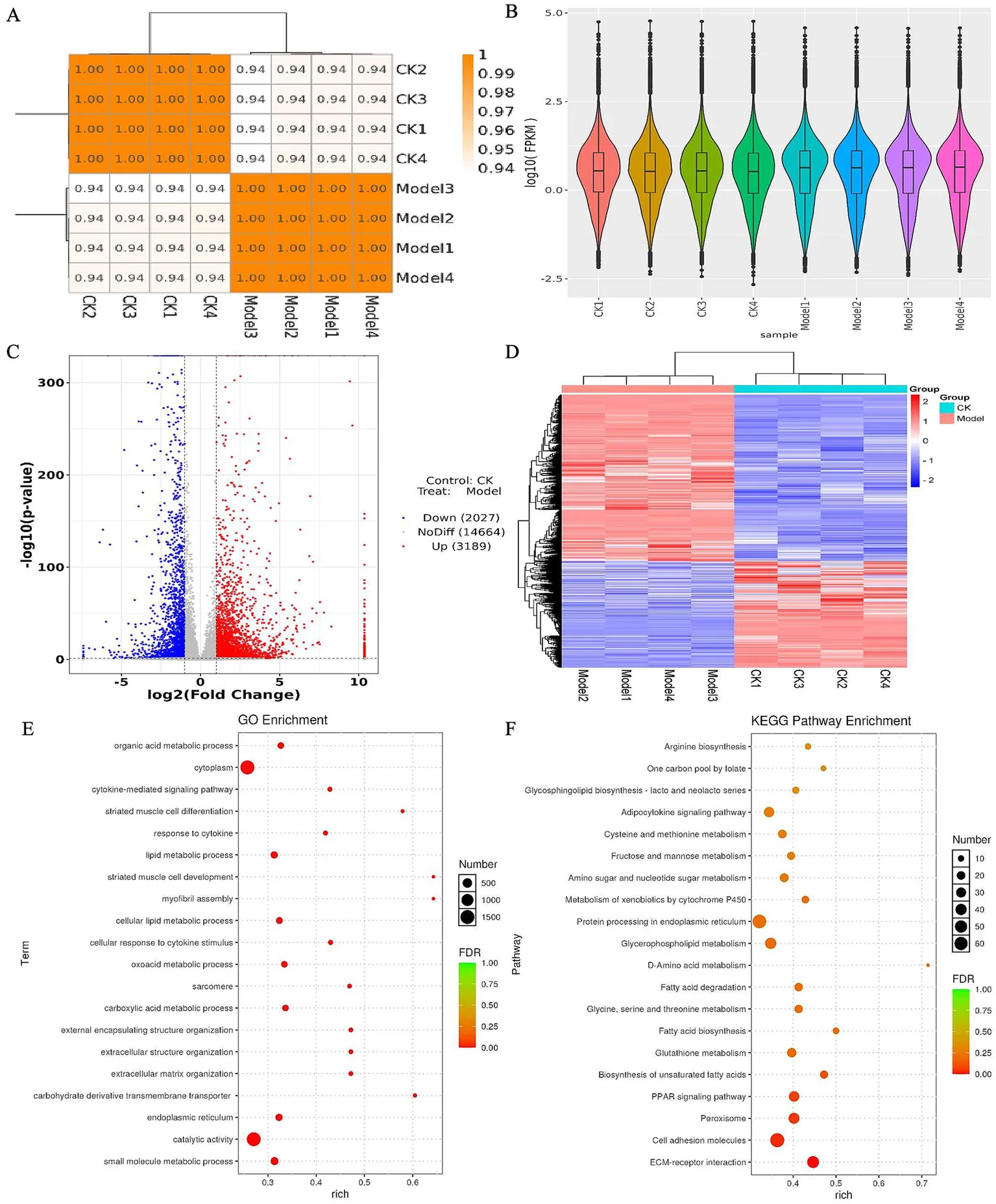
Transcriptomic analysis of intestinal responses to *Aeromonas hydrophila inf*ection in juvenile *Monopterus albus.* **(A)** Sample correlation heatmap between the CK and Model groups. **(B)** Distribution of gene expression levels based on log10(FPKM). **(C)** Volcano plot of differentially expressed genes between the CK and Model groups. Red dots represent upregulated genes, blue dots represent downregulated genes, and gray dots represent genes without significant differential expression. **(D)** Hierarchical clustering heatmap of differentially expressed genes. **(E)** GO enrichment analysis of differentially expressed genes. **(F)** KEGG pathway enrichment analysis of differentially expressed genes. CK: Uninfected control group; Model: *A. hydrophila-inf*ected group.

Compared with the CK group, a total of 5,216 differentially expressed genes were identified in the Model group, including 3,189 upregulated genes and 2,027 downregulated genes (Figure 6C). Hierarchical clustering analysis further showed that the CK and Model samples clustered separately, indicating that *A. hydrophila* infection induced extensive changes in intestinal gene expression patterns (Figure 6D).

GO enrichment analysis showed that these differentially expressed genes were mainly enriched in biological processes related to organic acid metabolism, cytokine-mediated signaling pathway, response to cytokine, lipid metabolic process, extracellular matrix organization, endoplasmic reticulum, and catalytic activity (Figure 6E). KEGG pathway enrichment analysis further revealed that the differentially expressed genes were mainly involved in arginine biosynthesis, adipocytokine signaling pathway, metabolism of xenobiotics by cytochrome P450, protein processing in the endoplasmic reticulum, fatty acid metabolism, glutathione metabolism, PPAR signaling pathway, peroxisome, cell adhesion molecules, and ECM–receptor interaction (Figure 6F). These results indicate that *A. hydrophila* infection triggered marked intestinal transcriptional remodeling in *M. albus*, particularly involving metabolism, inflammatory response, oxidative stress, endoplasmic reticulum stress, and intestinal barrier-related pathways.

### 3.8 PPI network analysis and qRT-PCR validation of hub genes

To further identify the key genes involved in the intestinal response of *M. albus* to *A. hydrophila* infection, a protein–protein interaction network was constructed based on the differentially expressed genes identified between the CK and Model groups. As shown in Figure 7A, the PPI network contained multiple highly connected nodes, suggesting that *A. hydrophila* infection induced coordinated changes in intestinal biological processes. According to the degree ranking, the top hub genes included *TP53*, *SRC*, *AKT1*, *GAPDH*, *STAT3*, *SIRT1*, *MET*, *JAK1*, *MTOR*, and *ERBB2* (Figure 7B). Among them, TP53 and SRC showed the highest degree values, indicating their potential central roles in the intestinal response to bacterial infection. The hub-gene interaction network further showed close associations among these genes, suggesting that they may participate in common regulatory processes related to inflammation, cell survival, metabolism, and stress responses (Figure 7C).

**Figure 7:**
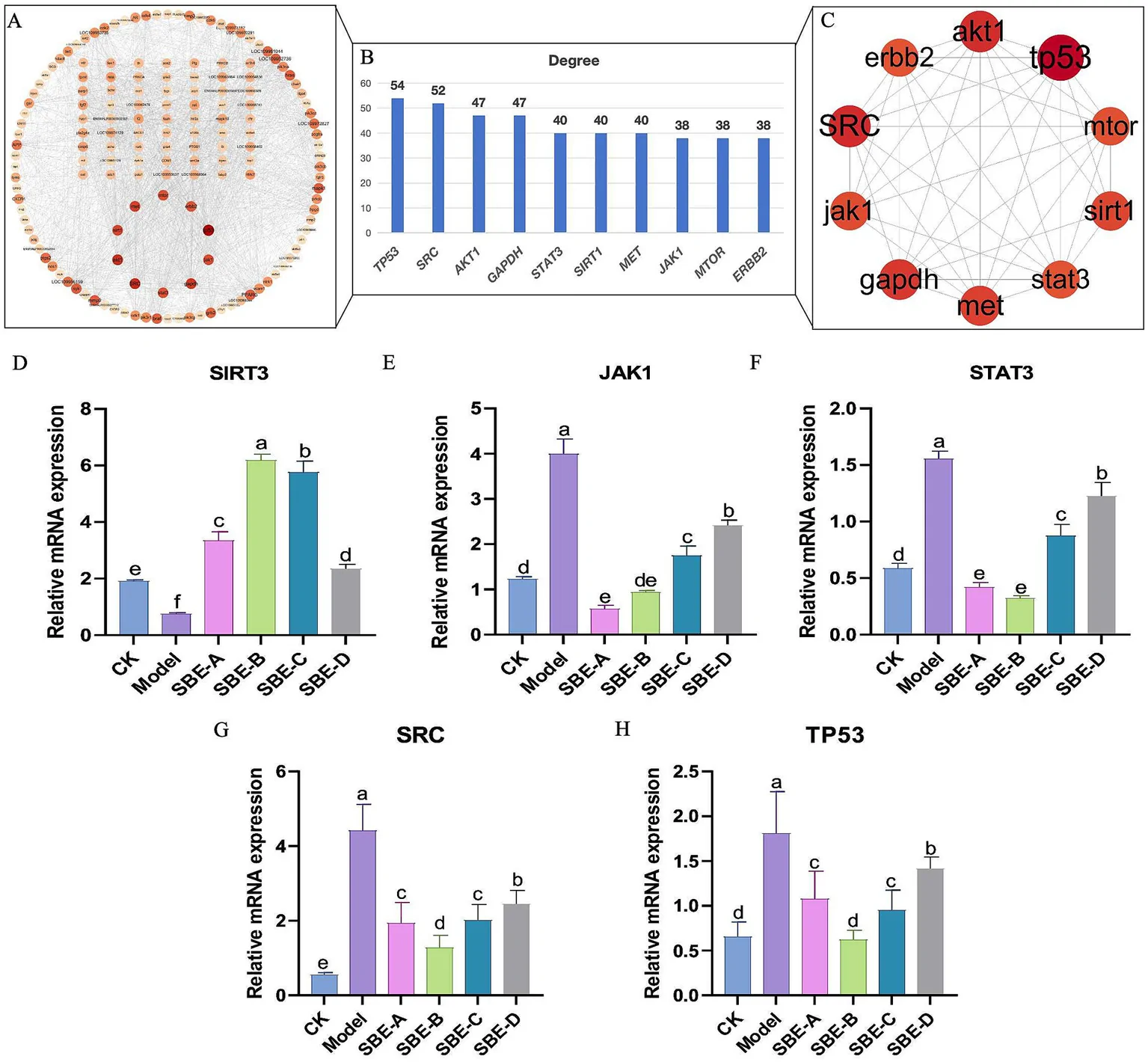
PPI network analysis and qRT-PCR validation of hub genes related to intestinal responses to *Aeromonas hydrophila inf*ection in juvenile *Monopterus albus*. **(A)** Protein–protein interaction (PPI) network of differentially expressed genes. **(B)** Degree ranking of the top 10 hub genes. **(C)** Interaction network of hub genes. **(D)** Relative mRNA expression of *SIRT3*. **(E)** Relative mRNA expression of *JAK1*. **(F)** Relative mRNA expression of *STAT3*. **(G)** Relative mRNA expression of *SRC*. **(H)** Relative mRNA expression of *TP53*. Values are expressed as mean ± SE. Different letters indicate significant differences among groups (*p* < 0.05). CK: Buninfected control group; Model: *A. hydrophila*-infected group; SBE-A: 0.5% SBE group after infection; SBE-B: 1% SBE group after infection; SBE-C: 2% SBE group after infection; SBE-D: 3% SBE group after infection.

To validate the transcriptomic results and evaluate the regulatory effect of SBE on these key genes, qRT-PCR was performed for selected hub genes and related regulatory genes. Compared with the CK group, *A. hydrophila* infection significantly decreased the mRNA expression of SIRT3, while markedly increasing the expression levels of *JAK1*, *STAT3*, *SRC*, and *TP53* in the Model group (*p* < 0.05; Figures 7D–H). Dietary SBE supplementation significantly reversed these infection-induced changes. These results indicate that dietary SBE supplementation may alleviate *A. hydrophila*-induced intestinal injury by regulating key genes associated with inflammatory signaling, cellular stress, and host defense responses.

### 3.9 Dietary SBE supplementation suppressed inflammatory and apoptosis-related gene expression after *A. hydrophila* infection

As shown in Figure 8, *A. hydrophila* infection markedly activated intestinal inflammatory and apoptosis-related gene expression in juvenile *M. albus*. Compared with the CK group, the Model group showed significantly increased mRNA expression levels of *NF-κB*, *TLR4*, *IL-1β*, *IL-6*, and *TNF-α* (*p* < 0.05; Figures 8A–E), indicating that bacterial infection induced a strong intestinal inflammatory response. However, dietary SBE supplementation significantly reduced the expression of these pro-inflammatory genes compared with the Model group (*p* < 0.05).

**Figure 8:**
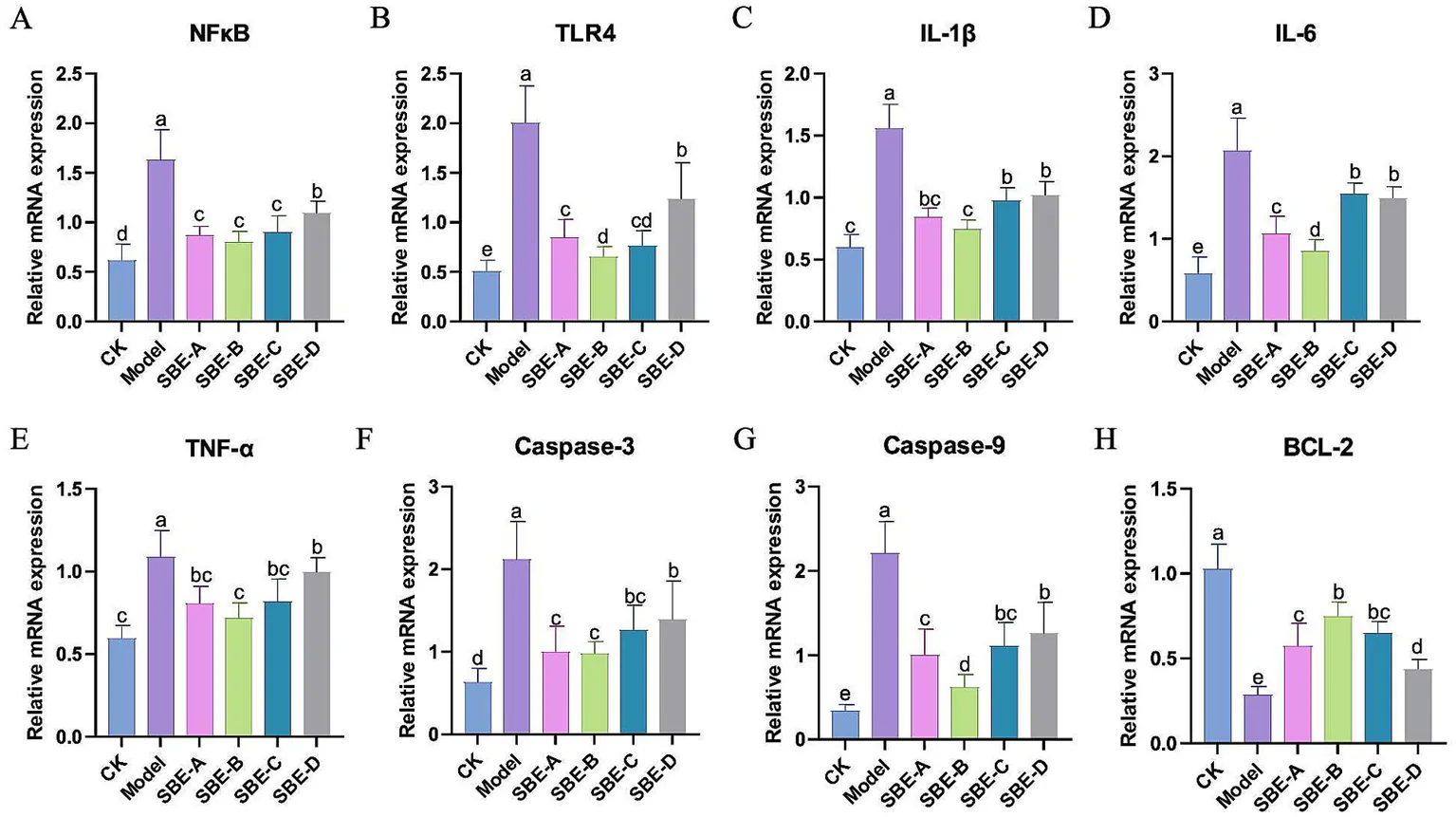
Effects of dietary SBE supplementation on inflammatory and apoptosis-related gene expression in the intestine of *M. albus* after *Aeromonas hydrophila* infection. **(A)** Relative mRNA expression of *NF-κB*; **(B)** Relative mRNA expression of *TLR4*; **(C)** Relative mRNA expression of *IL-1β*; **(D)** Relative mRNA expression of *IL-6*; **(E)** Relative mRNA expression of *TNF-α*; **(F)** Relative mRNA expression of *Caspase-3*; **(G)** Relative mRNA expression of *Caspase-9*; **(H)** Relative mRNA expression of *Bcl-2*. Values are expressed as mean ± SE. Different letters indicate significant differences among groups (*p* < 0.05). CK: Control group; Model: *A. hydrophila*-infected group; SBE-A: 0.5% SBE group; SBE-B: 1% SBE group; SBE-C: 2% SBE group; SBE-D: 3% SBE group.

The expression patterns of apoptosis-related genes were also affected by *A. hydrophila* infection and SBE supplementation. Compared with the CK group, the Model group showed significantly increased expression levels of *Caspase-3* and *Caspase-9* (*p* < 0.05), whereas Bcl-2 expression was significantly decreased (*p* < 0.05; Figures 8F–H), suggesting that bacterial infection promoted intestinal apoptosis. In contrast, SBE supplementation significantly downregulated *Caspase-3* and *Caspase-9* expression and partially restored *Bcl-2* expression compared with the Model group (*p* < 0.05). These results indicate that dietary SBE supplementation modulated inflammation- and apoptosis-related gene expression following *A. hydrophila* challenge.

## 4 Discussion

Previous studies have demonstrated that plant extracts contain a variety of bioactive compounds, including polysaccharides, flavonoids, alkaloids and essential oils, which can be used as functional feed additives in aquaculture by exerting beneficial effects such as appetite stimulation, growth promotion, antioxidant enhancement and immune regulation (Caderno et al., 2026; Salomón et al., 2020). As a commonly used plant-derived additive, *S. baicalensis* and its extracts have attracted extensive attention owing to their abundant flavonoids and multiple biological activities (Ni et al., 2026; Xia et al., 2023). In the present study, dietary supplementation with 0.5 and 1% SBE significantly improved FBW, WGR and SGR of juvenile *M. albus*, while markedly reducing FCR. These results indicated that appropriate SBE supplementation could improve feed utilization and production performance of *M. albus*. Similar findings have been reported in previous studies. Dietary supplementation with baicalein, one of the main bioactive compounds derived from *S. baicalensis*, enhanced the weight gain rate of koi carp and improved feed efficiency in tilapia (Li et al., 2023; Yin et al., 2021). Additionally, dietary SBE improved the specific growth rate and feed utilization of olive flounder (Cho et al., 2013), and the aerial part of *S. baicalensis* also promoted the growth and nutritive value of rabbit fish (Xia et al., 2021). Consistent with these studies, the present results further confirmed the growth-promoting potential of SBE in aquatic animals. However, the growth performance of *M. albus* was not further improved in the 2 and 3% SBE groups. This phenomenon may be associated with differences in extract source, effective component concentration, dietary inclusion level and species-specific responses (Kim et al., 2013). Therefore, the beneficial effect of SBE on growth performance may depend on an appropriate supplementation level rather than a simple dose-dependent pattern. It should also be noted that wheat bran content was adjusted to accommodate increasing SBE inclusion, resulting in small differences in crude protein and crude fiber among the experimental diets. Therefore, a contribution of these dietary compositional differences to the observed growth, physiological, and intestinal microbiota responses cannot be completely excluded. Future studies using nutritionally matched and isofibrous diets are needed to more precisely determine the specific effects of SBE. Notably, the lower FCR observed in the higher SBE groups was not accompanied by corresponding significant improvements in all growth-related indices. Because FCR is a ratio of feed intake to body-weight gain, this divergence should not be interpreted as evidence of metabolic energy reallocation. The present study did not evaluate nutrient digestibility, body composition, nutrient retention, or energy metabolism; therefore, the physiological basis of this non-parallel response remains unclear. Nevertheless, improved intestinal function may influence nutrient utilization efficiency and systemic metabolic allocation through the gut–metabolism–muscle axis. Nutritional interventions that maintain intestinal homeostasis have been suggested to affect tissue metabolism and nutrient partitioning (Yu and Li, 2025; Yu et al., 2025). However, muscle composition, energy metabolism, and flesh quality were not evaluated in the present study, and further investigations are required to clarify this potential interaction.

The improvement in growth performance may be closely related to the regulatory effect of SBE on intestinal structure and digestive function. The intestine is the main organ for nutrient digestion and absorption in fish and also serves as an important immune defense barrier (Guttuso et al., 2026; Liu et al., 2019). Maintaining intestinal structural integrity is therefore essential for sustaining growth and physiological homeostasis. In this study, dietary supplementation with SBE significantly increased intestinal villus length and improved villus arrangement, especially in the 0.5 and 1% SBE groups. The increase in villus length may enlarge the contact area between intestinal mucosa and nutrients, thereby promoting nutrient absorption and feed utilization (Zhang et al., 2023). In *M. albus*, previous studies have also demonstrated that dietary functional components can regulate intestinal structure and affect growth performance (Deng et al., 2023; Yang et al., 2024). These findings suggested that the growth-promoting effect of SBE may be partly attributed to the improvement of intestinal morphology and absorptive capacity. However, excessive supplementation did not further improve villus development, suggesting that high-dose SBE may weaken its beneficial effect on intestinal structure.

Fish are frequently exposed to various stressors during intensive aquaculture, which may lead to excessive production of reactive oxygen species and oxidative damage (Chen et al., 2025; Chukwuma et al., 2025). To counteract oxidative stress, fish have developed a relatively complete antioxidant defense system, including enzymatic antioxidants such as SOD and CAT, and non-enzymatic antioxidants such as GSH, vitamin C and vitamin E (Olsvik et al., 2005). In the present study, dietary SBE significantly enhanced intestinal GSH, SOD and T-AOC levels in *M. albus*, indicating that SBE improved intestinal antioxidant capacity. Meanwhile, the mRNA expression levels of antioxidant-related genes, including *CAT*, *GSH*, *SOD* and *Keap1*, were significantly upregulated in the 1% SBE group. These results suggested that SBE enhanced antioxidant defense at both biochemical and transcriptional levels. Similar effects have been reported in other aquatic animals. Dietary baicalin improved antioxidative status and protected GIFT tilapia from oxidative stress-induced liver injury (Jia et al., 2021), while dietary baicalein increased antioxidant activity in koi carp (Du et al., 2022). The antioxidant effect of SBE may be attributed to its abundant flavonoid components, since flavonoids are well known for their strong free radical scavenging ability (Afanas'ev et al., 1989; Mutha et al., 2021). Previous studies have also demonstrated that dietary flavonoids, such as baicalein, can enhance antioxidant capacity and intestinal health in aquatic animals, supporting the potential contribution of flavonoid components to the protective effects of SBE (Du et al., 2022).

MDA is a typical end product of lipid peroxidation and is widely used as an indicator of oxidative damage in biological tissues (Tsikas, 2017). In this study, dietary SBE significantly reduced intestinal MDA content in *M. albus*, indicating that SBE effectively alleviated lipid peroxidation. Similar results have been observed in broiler chickens, grass carp and yellow catfish, in which *S. baicalensis* extract, baicalein or baicalin reduced MDA levels and improved antioxidant status (Xu et al., 2019). In particular, baicalin was reported to mitigate *A. hydrophila*-induced lipid peroxidation and antioxidant imbalance in yellow catfish (Yan et al., 2024). These findings were consistent with the present study and suggested that SBE could protect intestinal tissues against oxidative damage. However, the antioxidant-promoting effect of SBE in *M. albus* was not dose-dependent. Compared with the 1% SBE group, the high-dose groups showed weaker antioxidant responses. Previous studies have suggested that excessive flavonoid intake may exert potential toxic effects by participating in redox reactions and promoting ROS generation under certain conditions (Galati and O'Brien, 2004; Tang and Zhang, 2021). The basis for this non-linear dose–response cannot be determined from the present study because specific toxicological, serum biochemical, or metabolic endpoints were not evaluated. Therefore, the present findings only indicate that increasing the dietary SBE inclusion level above 1% did not produce additional beneficial effects under the experimental conditions.

In addition to oxidative stress regulation, SBE also affected intestinal immune-related gene expression. IFN-γ is an important pro-inflammatory cytokine, and increased IFN-γ expression is usually associated with local inflammatory responses (Jorgovanovic et al., 2020). In the present study, dietary supplementation with 0.5 and 1% SBE significantly reduced intestinal *IFN-γ* expression, suggesting that appropriate SBE supplementation may alleviate basal intestinal inflammatory status and improve immune homeostasis. Similar findings have been reported in yellow catfish, where baicalein treatment alleviated intestinal inflammatory infiltration induced by *A. hydrophila* infection and reduced *IFN-γ* expression in immune tissues (Yan et al., 2024). These results suggested that SBE may not only enhance antioxidant defense but also contribute to the regulation of intestinal immune function. However, IFN-γ expression was higher in the 3% SBE group than in the lower-dose SBE groups, further indicating a non-linear response to increasing SBE inclusion. The biological basis of this response remains unclear. Overall, among the inclusion levels examined, 1% SBE produced the most consistently favorable response under the present experimental conditions.

In recent years, the relationship between intestinal microbiota and host health in fish has received increasing attention because of its important roles in nutrient metabolism, immune regulation and disease resistance (Medina-Félix et al., 2023; Pérez et al., 2010). Dietary plant extracts or their active components can regulate intestinal microbiota and improve microbial stability in aquatic animals (Blaufuss et al., 2020; Pulgar et al., 2021). In this study, dietary supplementation with SBE significantly increased Chao1 and Shannon indices in the 1% SBE group, indicating that appropriate SBE supplementation enhanced intestinal microbial richness and diversity. Beta diversity analysis further showed that the intestinal microbial community structure differed between the CK group and SBE-supplemented groups. Similar regulatory effects have been reported in previous studies. Baicalein supplementation increased intestinal microbial diversity in broiler chickens and improved intestinal flora in koi carp (Li et al., 2023; Liu et al., 2025). Therefore, the present results suggested that dietary SBE may promote intestinal microbial stability in *M. albus*, although its effect may vary according to supplementation level and animal species.

At the phylum level, *Proteobacteria*, *Firmicutes* and *Bacteroidota* were among the dominant bacterial phyla in the intestine of *M. albus*. These phyla are commonly detected in fish intestinal microbiota and are closely related to nutrient metabolism and host health (Cornuault et al., 2022; Ye et al., 2014). In this study, dietary SBE supplementation significantly changed their relative abundance. Specifically, 0.5 and 1% SBE increased the relative abundance of *Firmicutes* and *Bacteroidota*, while decreasing the relative abundance of *Proteobacteria* and the Firmicutes/Bacteroidota ratio. *Firmicutes* and *Bacteroidota* are considered important bacterial groups involved in nutrient digestion, energy metabolism and immune regulation (Wang et al., 2022). However, an abnormal increase in the Firmicutes/Bacteroidota ratio has been associated with metabolic disorders and chronic inflammatory status (Turnbaugh et al., 2006). Therefore, the reduced Firmicutes/Bacteroidota ratio in the SBE-supplemented groups suggested that SBE may contribute to maintaining intestinal metabolic balance. Moreover, Proteobacteria contains many opportunistic pathogens, and its abnormal expansion is often regarded as a microbial signature of epithelial dysfunction and dysbiosis (Litvak et al., 2017). The significant reduction in Proteobacteria abundance indicated that SBE supplementation may help prevent intestinal microbial imbalance and epithelial inflammation in *M. albus*.

At the genus level, dietary SBE supplementation significantly reduced the relative abundance of *Plesiomonas*, *Brevinema* and *Pelomonas*, while increasing the relative abundance of *Cetobacterium*. Previous studies have suggested that *Plesiomonas* and *Brevinema* may be associated with intestinal inflammation, microbial imbalance and pathogenic risk in fish (Liu et al., 2024; Wu et al., 2023). Therefore, the reduction of these potential pathogenic bacteria indicated that SBE may help inhibit harmful bacterial colonization in the intestine. In contrast, *Cetobacterium* is a common dominant bacterial genus in freshwater fish and is considered beneficial for host health through nutrient metabolism, vitamin production and inhibition of pathogen colonization (Paimeeka et al., 2024; Zhang et al., 2022). The increase in *Cetobacterium* abundance may therefore contribute to improved intestinal function and disease resistance in *M. albus*. Similar results have been reported in koi carp, where dietary baicalein inhibited intestinal pathogenic microorganisms and promoted the proliferation of beneficial bacteria (Qiu et al., 2025). These findings suggested that SBE may maintain intestinal health by promoting probiotic colonization and reducing the abundance of potential pathogens.

*A. hydrophila* is an important opportunistic pathogen in aquaculture and can cause intestinal inflammation, septicemia, tissue damage and high mortality in fish (Guo et al., 2024). In this study, *A. hydrophila* challenge sharply decreased the survival of the Model group, whereas dietary SBE supplementation improved survival in infected *M. albus.* Moreover, SBE supplementation significantly reduced the culturable bacterial burden in the intestine, spleen, and liver, particularly in the 1% SBE group. Together, these findings support an enhanced resistance of *M. albus* to *A. hydrophila* challenge following SBE supplementation. However, because LB agar is non-selective and individual colonies were not confirmed at the species level, the plate-count data should be interpreted as culturable bacterial burden rather than direct species-specific quantification of *A. hydrophila*. Similar protective effects have been reported in olive flounder and white shrimp, where dietary SBE enhanced challenge resistance against pathogenic bacteria (Cho et al., 2013). These findings suggested that the protective effect of SBE against bacterial infection may be achieved through the combined regulation of antioxidant capacity, intestinal microbiota and immune defense. It should be noted that the challenge dose used in the present study produced a severe acute lethal infection, with survival in the Model group reaching 0% by 60 h. Therefore, the protective effects observed under this stringent challenge condition should not be directly extrapolated to natural or less severe infection. In addition, bacterial burden was assessed only at 24 h post-infection to characterize early dissemination before extensive mortality. Consequently, the present study does not define the complete temporal dynamics of bacterial colonization and clearance, and future studies should include multiple post-infection time points and dose-ranging challenge experiments.

The intestinal barrier is an important defense line against pathogen invasion (Chen et al., 2021). Tight junction proteins, including ZO-1, Claudin-1 and Occludin, are key structural components that maintain epithelial barrier integrity (Wang et al., 2023). In the present study, *A. hydrophila* infection significantly downregulated the expression of *ZO-1*, *Claudin-1* and *Occludin*, suggesting that bacterial infection impaired intestinal barrier function. However, dietary SBE supplementation restored the expression of these tight junction-related genes, especially in the 1% SBE group. These results indicated that SBE may protect the intestinal barrier and reduce bacterial translocation. Similar findings have been reported in fish intestinal injury models, where functional additives improved mucosal barrier function and reduced inflammatory damage (Jiang et al., 2019; Zheng et al., 2022). Combined with the decreased culturable bacterial burdens observed in the SBE groups, these results suggested that modulation of tight junction-related gene expression may be associated with the improved resistance observed after SBE supplementation. However, because the corresponding proteins were not directly quantified, these transcriptional changes cannot by themselves demonstrate restoration of physical intestinal barrier integrity. Therefore, the present findings should be interpreted as evidence that SBE modulated tight junction-related gene expression following infection.

Transcriptomic analysis was further conducted to explore the intestinal response of *M. albus* to *A. hydrophila* infection. A total of 5,216 DEGs were identified between the CK and Model groups, including 3,189 upregulated genes and 2,027 downregulated genes. GO and KEGG enrichment analyses showed that these DEGs were mainly involved in cytokine-mediated signaling pathway, lipid metabolic process, extracellular matrix organization, protein processing in the endoplasmic reticulum, glutathione metabolism, PPAR signaling pathway, cell adhesion molecules and ECM–receptor interaction. These pathways are closely associated with inflammation, oxidative stress, epithelial structure and metabolic regulation. The enrichment of glutathione metabolism was consistent with the antioxidant changes observed in this study, while the enrichment of cell adhesion molecules and ECM–receptor interaction was consistent with the changes in tight junction-related gene expression. However, transcriptome sequencing was performed only between the CK and Model groups. Therefore, the transcriptomic results mainly reflected the intestinal molecular response to *A. hydrophila* infection and provided a basis for subsequent hub gene screening, rather than directly proving the global transcriptomic regulation of SBE.

Based on the DEGs, PPI network analysis identified several hub genes, including *TP53*, *SRC*, *AKT1*, *GAPDH*, *STAT3*, *SIRT1*, *MET*, *JAK1*, *MTOR* and *ERBB2*. These genes are closely associated with inflammatory responses, apoptosis, metabolism and cellular stress. qRT-PCR validation showed that *A. hydrophila* infection significantly increased the expression levels of *JAK1*, *STAT3*, *SRC* and *TP53*, while decreasing *SIRT1* expression. Dietary SBE supplementation reversed these infection-induced changes, especially in the 1% SBE group. JAK/STAT signaling plays an important role in cytokine-mediated immune responses, while *TP53* is involved in stress-induced apoptosis (O'Shea and Plenge, 2012; Ogawa et al., 2025). SIRT1 is a key regulator of inflammation and metabolism and can inhibit NF-κB activity through the deacetylation of p65 (Xie et al., 2013). These findings indicate that SBE supplementation was associated with changes in the transcription of several infection-responsive hub genes involved in inflammatory and stress-related processes. However, the transcriptomic analysis itself did not include SBE-treated groups, and the subsequent validation was performed at the mRNA level. Therefore, these genes should be considered candidate SBE-responsive targets rather than confirmed molecular mediators. Further studies are required to verify their roles at the protein and functional levels. In particular, Western blotting and other protein-based approaches are necessary to determine whether the observed transcriptional changes are accompanied by corresponding alterations in protein abundance and pathway activation.

Inflammation is one of the major pathological responses induced by bacterial infection (Bäumler and Sperandio, 2016). In the present study, *A. hydrophila* infection significantly upregulated the expression levels of *TLR4*, *NF-κB*, *IL-1β*, *IL-6* and *TNF-α*, indicating the activation of intestinal inflammatory responses. TLR-mediated signaling is an important mechanism by which fish recognize bacterial components and activate downstream inflammatory pathways (Sabroe et al., 2008). NF-κB is a key transcription factor involved in the regulation of pro-inflammatory cytokines, and its activation can promote the expression of *IL-1β*, *IL-6* and *TNF-α* (Yu H. et al., 2020). Dietary SBE supplementation significantly reduced the expression of these inflammatory genes, suggesting that SBE alleviated *A. hydrophila*-induced intestinal inflammation. Similar results have been reported in tilapia, where baicalin inhibited oxidative stress-induced inflammatory gene expression (Yu et al., 2021). Baicalin also alleviated LPS-induced oxidative stress and inflammation through NF-κB-related signaling pathways in intestinal epithelial cells (Fu et al., 2021). These results indicated that SBE may exert anti-inflammatory effects by suppressing TLR4/NF-κB-related inflammatory signaling. Similar regulatory effects of plant-derived extracts on intestinal inflammatory homeostasis through TLR4/NF-κB-associated pathways have also been reported (Yu Z. et al., 2020).

Apoptosis is another important process involved in pathogen-induced tissue injury (Liu et al., 2022). In this study, *A. hydrophila* infection significantly increased the mRNA expression of *Caspase-3* and *Caspase-9* and decreased *Bcl-2* expression, indicating an enhanced apoptosis-related transcriptional response. Dietary SBE supplementation reduced *Caspase-3* and *Caspase-9* mRNA expression and increased *Bcl-2* expression relative to the Model group. These findings indicate that SBE modulated apoptosis-related gene expression following infection. However, because apoptosis was not directly evaluated at the protein or cellular level, these transcriptional changes should not be interpreted as direct evidence of inhibition of apoptosis. Previous studies have shown that baicalein and baicalin can alleviate tissue injury by reducing oxidative stress, inflammation and cell apoptosis (You et al., 2025). Therefore, the anti-apoptotic effect of SBE may cooperate with its antioxidant, anti-inflammatory and microbiota-regulating effects to maintain intestinal homeostasis and improve survival after *A. hydrophila* challenge.

Overall, the present findings indicate that dietary SBE supplementation was associated with improved growth performance, intestinal antioxidant capacity, intestinal morphology, microbiota composition, and resistance to *A. hydrophila* challenge in juvenile *M. albus*. Among the inclusion levels tested, the 1% SBE group showed the most consistently favorable responses under the present experimental conditions. However, the underlying mechanisms should be interpreted cautiously because most molecular indicators were evaluated only at the mRNA level. Further studies incorporating protein-level validation, metabolite analysis, and microbiota functional assessment are needed. In addition, primer amplification efficiencies and standard-curve parameters were not determined in the original qRT-PCR experiments, which represents a methodological limitation.

## 5 Conclusion

In conclusion, dietary supplementation with *Scutellaria baicalensis* extract improved growth performance, intestinal antioxidant capacity, intestinal morphology, and intestinal microbiota stability in juvenile *Monopterus albus*. Among the dietary inclusion levels examined in the present study, 1% SBE produced the most consistently favorable responses under the experimental conditions. Following *Aeromonas hydrophila* challenge, SBE supplementation improved survival, reduced culturable bacterial burdens in the intestine, spleen, and liver, and modulated tight junction-, inflammation-, and apoptosis-related gene expression. Transcriptomic and PPI analyses of the CK and Model groups further characterized the intestinal molecular responses associated with *A. hydrophila* infection rather than directly demonstrating transcriptome-wide regulation by SBE. Overall, these findings support the potential of SBE as a functional feed additive for juvenile *M. albus*. However, further dose–response studies using nutritionally matched diets and different culture conditions are required before an optimal dietary inclusion level can be established for practical aquaculture.

## Data Availability

The raw 16S rRNA amplicon sequencing and RNA-seq data have been deposited in the NCBI Sequence Read Archive under BioProject accession numbers PRJNA1526581 and PRJNA1526701, respectively.
