# Supplementary material for: Dietary Scutellaria baicalensis extract enhances growth, intestinal health, and resistance to Aeromonas hydrophila in juvenile Asian swamp eel (Monopterus albus)
Source: Front Microbiol. 2026 Sep 18;17:1940498. doi: 10.3389/fmicb.2026.1940498 (PMC13631943; doi:10.3389/fmicb.2026.1940498)
Supplement: Supplementary file 1 [file Supplementary_file_1.DOCX]

## **Supplementary Figure 1.** Extended analysis of intestinal microbiota in juvenile Monopterus albus after dietary SBE supplementation.

(A) Principal coordinate analysis (PCoA) plot. (B) Non-metric multidimensional scaling (NMDS) plot. (C) Sample correlation heatmap. (D) Differential microbial taxa between the CK group and SBE-supplemented groups. (E) Functional composition of the intestinal microbiota. (F) Heatmap of predicted microbial functions. (G) Microbial co-occurrence network. CK: basal diet group; SBE-A: 0.5% SBE group; SBE-B: 1% SBE group; SBE-C: 2% SBE group; SBE-D: 3% SBE group.
